# Comparison of nicotine exposure during pregnancy when smoking and abstinent with nicotine replacement therapy: systematic review and meta‐analysis

**DOI:** 10.1111/add.14473

**Published:** 2018-12-11

**Authors:** Charlotte Hickson, Sarah Lewis, Katarzyna Anna Campbell, Sue Cooper, Ivan Berlin, Ravinder Claire, Cheryl Oncken, Tom Coleman‐Haynes, Tim Coleman

**Affiliations:** ^1^ Division of Primary Care University of Nottingham Nottingham UK; ^2^ Division of Epidemiology and Public Health University of Nottingham Nottingham UK; ^3^ Sorbonne Université Faculté de médecine‐Hôpital Pitié‐Salpêtrière Paris France; ^4^ University of Connecticut School of Medicine Farmington CT USA

**Keywords:** Cotinine, nicotine, nicotine replacement therapy, pregnancy, smoking, smoking cessation

## Abstract

**Background and aims:**

Smoking during pregnancy is strongly associated with negative pregnancy and perinatal outcomes. Some guidelines recommend nicotine replacement therapy (NRT) for smoking cessation during pregnancy, but adherence with NRT is generally poor and could be partially explained by nicotine‐related safety concerns. We compared pregnant women's cotinine and nicotine exposures from smoking with those when they were abstinent from smoking and using NRT.

**Design:**

Systematic review with meta‐analysis and narrative reporting. Twelve studies were included: in most, only one type of NRT was used. Seven were quality‐assessed and judge of variable quality.

**Setting:**

Studies from any setting that reported nicotine or cotinine levels when smoking and later when abstinent and using NRT.

**Participants:**

Pregnant women who smoked and became abstinent but used NRT either in a cessation study or in a study investigating other impacts of NRT.

**Measurements:**

We quality‐assessed longitudinal cohort studies using a modified version of the Newcastle–Ottawa scale. For meta‐analysis, we used mean within‐person differences in cotinine or nicotine levels when smoking and at later follow‐up when abstinent and using NRT. Where such data were not available, we calculated differences in group mean levels and reported these narratively, indicating where data were not completely longitudinal.

**Findings:**

Of the 12 included studies, four cotinine‐measuring studies (*n* = 83) were combined in a random effects meta‐analysis; the pooled estimate for the mean difference (95% confidence intervals) in cotinine levels between when women were smoking and abstinent but using NRT was 75.3 (57.1 to 93.4) ng/ml (*I* ^2^ = 42.1%, *P* = 0.11). Of eight narratively‐described studies, six reported lower cotinine and/or nicotine levels when abstinent and using NRT; two had mixed findings, with higher levels when abstinent but using NRT reported from at least one assay time‐point.

**Conclusions:**

Pregnant women who use nicotine replacement therapy instead of smoking reduce their nicotine exposure.

## Introduction

Smoking in pregnancy causes much morbidity and mortality 1 and rates are highest among younger, socially disadvantaged women 2. Forty per cent of socio‐economic inequalities in stillbirths and infant deaths are smoking‐related 3, and smokers’ children are twice as likely to become smokers themselves 4; however, this is all avoidable. Stopping smoking in pregnancy improves birth outcomes 5; permanent cessation after pregnancy improves women's health and may also improve their children's health by diminishing second‐hand smoke exposure and possibly by reducing penetration of smoking across the generations 6.

In the United Kingdom, when other cessation methods have been ineffective, pregnant women who want to stop can be recommended to use nicotine replacement therapy (NRT) 7 and guidance developed for use across the European Union (EU) takes the same approach 8. All UK stop smoking services (SSS) offer NRT to pregnant smokers 9, and 11% of UK pregnant smokers receive NRT prescriptions 10. Although NRT is effective outside pregnancy and the risk ratio (RR) [95% confidence interval (CI)] for cessation using NRT in non‐pregnant smokers is 1.60 (1.53–1.68) 11, in pregnancy NRT has, at best, only borderline effectiveness for promoting smoking cessation. From all trials of NRT in pregnancy, the risk ratio (95% CI) for cessation with NRT in pregnancy is 1.43 (1.03–1.93), but when meta‐analysis is restricted to include only least‐biased, placebo randomized controlled trials (RCTs), there is less evidence that NRT works and the risk ratio (RR) is reduced further (RR 1.28, 95% CI = 0.99–1.66) 12. One of the most plausible explanations for NRT appearing less effective in pregnancy is that pregnant women may not use NRT for long enough or in sufficient doses for it to be effective. For example, of UK pregnant smokers who are offered or prescribed NRT, 70% receive only a 2‐week supply 10. Similarly, in some trials which have enrolled pregnant smokers, only 7–30% of participants completed recommended courses of NRT 12. In contrast, non‐pregnant smokers enrolled into cessation trials adhere more strongly, using up to 94% of their intended NRT treatment courses 13.

Improving pregnant smokers’ adherence to NRT could result in this being more effective at helping them to stop smoking. In non‐pregnant smokers, prescribing higher doses of NRT results in greater use of NRT 13, and this greater use of NRT is causally associated with successfully stopping smoking 13, 14. There is very little similar research in pregnancy; however, we know that the rate of nicotine metabolism is substantially accelerated in pregnancy 15, 16. This means that any given dose of NRT generates lower blood nicotine concentrations than the same dose used either before pregnancy or in the postpartum period. It is also known that, in pregnancy, faster nicotine metabolism is associated with lower cessation rates 17, possibly because pregnant NRT users have more rapid nicotine turnover and so will experience stronger nicotine withdrawal symptoms, be more likely to perceive NRT as unhelpful and stop using it. One would therefore only expect NRT to be as effective during pregnancy as it is either before or afterwards if pregnant women's adherence levels were improved such that they obtained sufficient nicotine to ameliorate the impact of increased metabolism.

Pregnant women's reluctance to use NRT seems to be partially explained by their worries about the safety of nicotine 18. However, as NRT contains none of the harmful products of tobacco combustion there has long been consensus that, for pregnant women, NRT is probably safer than smoking 19. Nevertheless, as we cannot be completely sure that nicotine is entirely safe in pregnancy, women probably need reassurance. Hence, to help pregnant women to decide about using NRT, clear information about nicotine exposures generated when smoking or using NRT could be useful. Such information could also assist health professionals who counsel pregnant women about using NRT. In this review, therefore, we aimed to identify and describe studies which report nicotine or cotinine levels in pregnant women when smoking and subsequently when abstinent from smoking and using NRT, comparing these to estimate any differences between body fluid concentrations. A secondary aim was to investigate how any differences might be influenced by type(s) of NRT used or health professionals’ instructions on how NRT should be used.

## Method

This review followed the Preferred Reporting Items for Systematic Reviews and Meta‐Analyses (PRISMA) methods 20. A review protocol has been published 21. To be included, papers needed to study pregnant women who smoke and who subsequently became abstinent while using NRT. Studies had to report the same women's nicotine or cotinine body fluid levels both when smoking and when using NRT. The design had to either be longitudinal or have a design which implied that longitudinal data might be available, even if such data were not reported in study publications (e.g. from NRT‐allocated arms in RCTs of NRT).

### Searches

We developed a search strategy in MEDLINE using a combination of MESH and plain text terms and adapted it to use in Web of Science and EBSCO (see Supporting information, Appendix S1); the strategy was optimized against its ability to find three studies which we knew should be included in the final review. Searches of these three platforms allowed access to six databases: MEDLINE, EMBASE (Excerpta Medica Database), PsycINFO, MIDRIS (Maternity and Infant Care Database), SSCI (Social Sciences Citation Index) and CINHAL (Cumulative Index to Nursing and Allied Health Literature), and were completed by 29 August 2017. We also searched GSK clinical trials (https://www.gsk‐clinicalstudyregister.com/); World Health Organization International Clinical Trials Registry Platform (www.who.int/trialsearch); US National Library of Medicine Clinical Trials database (clinicaltrials.gov/); and the ISRCTN registry (http://www.isrctn.com/). Finally, we searched the Cochrane Library using the terms ‘smoking’, ‘pregnancy’ and ‘nicotine replacement’. Non‐bibliographic database searches were completed by 7 September 2017. There were no language restrictions and literature was searched from 1980, as the first trials of NRT were reported after that. In tandem with electronic searches, we scanned the references of papers included in reviews identified by the searches, and which covered the topic of interest, but were not eligible for inclusion.

### Study selection

Identified citations (titles and abstracts) were manipulated in an EndNote library. One reviewer (C.H.) screened these to assess whether or not articles should be included, and where there was uncertainty or papers were thought likely to be eligible, full texts were assessed by two reviewers with agreement on inclusion or exclusion being reached by consensus.

### Data extraction

Data were extracted by one researcher (C.H.) and checked by a second (T.C.). The following study details were extracted: objectives, setting, inclusion and exclusion criteria, study design and analysis; and number and characteristics of participants providing data for this review, baseline information on nicotine addiction or heaviness of smoking. The following intervention details were extracted: completeness of follow‐up for women in longitudinal analyses; reasons for dropout; biochemical confirmation of participant's smoking abstinence or not; dose(s) and type(s) of NRT used; instructions given on how regularly and for how long NRT should be used. The following details on measurements were extracted: body fluids sampled; whether nicotine or cotinine was assayed; time‐points at which samples were taken and timings of samples relative to smoking or NRT use; and relevant numerical findings (e.g. mean differences between concentrations of cotinine or nicotine concentrations at baseline and later time‐points). For ongoing studies, we e‐mailed the Principal Investigator enquiring whether data were available and we asked the same of corresponding authors for those papers which reported insufficient data for meta‐analysis (see ‘Analysis’ below). For two studies 22, 23 we converted graphical data to numerical using WebPlotDigitizer software 24.

### Risk of bias assessment

We quality‐assessed those studies which had been designed as longitudinal cohort studies and which stated, a priori, that a reason for the study was to take measurements when smoking and later abstinent and using NRT. These studies designs were, therefore, directly relevant to this review—any biases in methods used could be adjudged directly from published reports; this was performed using Wells’ modified version of the Newcastle–Ottawa Scale (NCOS) 25 (see Supporting information, Appendix S2). Papers were independently rated by two researchers, ratings were compared and disagreements resolved by discussion. We did not quality‐assess studies which had not been designed as before–after longitudinal studies (e.g. RCTs or secondary analysis of RCTs). For these studies, as studies’ data were not being used in a manner consistent with their designs (e.g. data from RCT arms treated as cohorts), the quality of the original study would not necessarily be relevant to review analyses. Similarly, where authors provided additional, unpublished data, we did not attempt quality assessment.

### Modifications to the Newcastle–Ottawa scale

Wells’ modified version of the NCOS allocates stars to reflect study quality on eight items grouped under three domains: selection or comparability of study group and ascertainment of exposure/outcome 25. We did not use three NCOS items and amended others, such that the maximum score was seven stars. Two items attracted up to two stars (‘representativeness of cohort’ and ‘adequacy of cohort follow‐up’) and one star for the remaining three (ascertainment of exposures, method for confirming abstinence and appropriateness of sample timing). We did not use the item ‘Selection of the non‐exposed cohort’, as included studies compared measurements from the same women at different times and did not have non‐exposed controls. ‘Demonstration that outcome of interest was not present at start of study’ was irrelevant, as all studies measured outcomes (e.g. cotinine) and ‘Comparability of cohorts on the basis of the design or analysis’ was not discriminatory, as all studies were longitudinal cohorts. All five items and scoring are fully described in Supporting information, Appendix S2.

### Analysis

Longitudinal, within‐person data, from the same women at baseline and at later time‐points, were used to estimate the mean differences between body fluid levels of nicotine or cotinine when smoking and later when abstinent and using NRT. We aimed to provide a pooled estimate of this difference in body fluid levels and to investigate the impacts of the type and dose of NRT and gestational age, but anticipated that the meta‐analysis undertaken would depend upon the available data and that a final decision on which studies (if any) to include in analyses would be taken once available literature were identified. For inclusion in meta‐analyses, study manuscripts had to report such a mean difference and its standard error or to report sufficient other data from which these could be calculated. Where such data were not included in papers, we contacted authors requesting either aggregated data as mean differences and standard errors or as individual participants’ data. A saliva : blood cotinine ratio has been reported as 1.01 (95% CI = 0.99–1.04) 26, so blood and saliva cotinine levels were considered interchangeable; nicotine and cotinine values and also urinary and saliva cotinine readings are not interchangeable, so these data were not aggregated.

Meta‐analysis was conducted in Stata version 15 using the Metan command employing random‐effects models 27 to provide a pooled, weighted estimate for the mean difference in cotinine levels when smoking and later when abstinent and using NRT 28. Two studies reported independent cohorts of women who had received different types or combinations or NRT 29, 30. As we anticipated that there would potentially be more variation between cohorts reported within one study, exposed to different types, doses or combinations of NRT than between cohorts reported in different studies, we treated such cohorts as independent studies in the random‐effects meta‐analysis. Heterogeneity was assessed using the *I* ^2^ statistic 31.

For studies which could not be included in meta‐analysis, we calculated differences in group mean levels (of cotinine or nicotine) and report these narratively, indicating where data were not completely longitudinal. For studies which provided ‘within‐participant’, longitudinal data with no loss to follow‐up, percentage nicotine substitution was calculated by dividing follow‐up mean cotinine (nicotine) levels by baseline ones and multiplying by 100. The percentage nicotine substitution measure indicates how completely NRT substitutes for nicotine from smoked tobacco.

## Results

**Figure 1:**
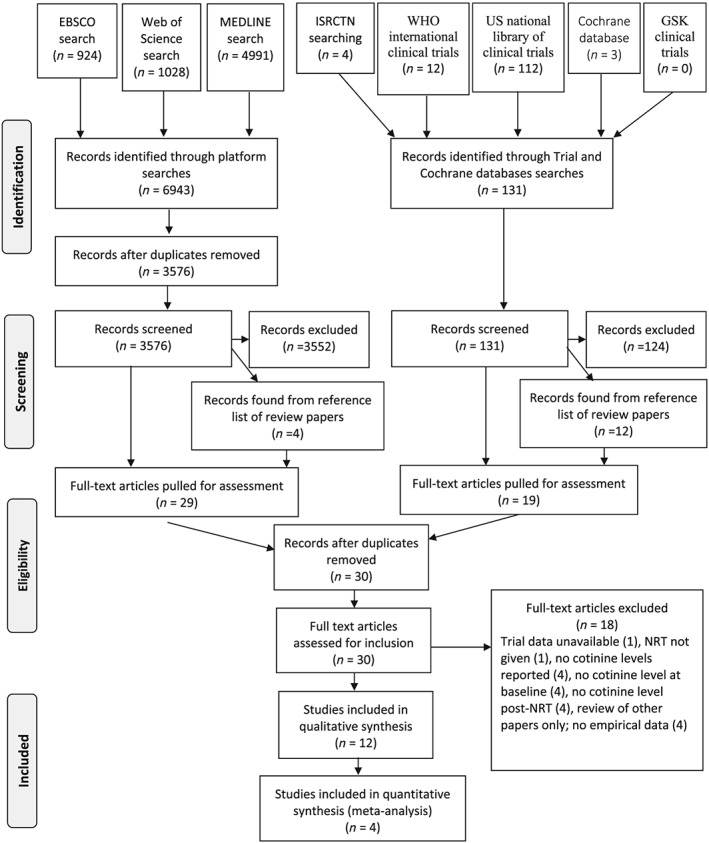
Preferred Reporting Items for Systematic Reviews and Meta‐Analyses (PRISMA) diagram: study selection. *From*: Moher D, Liberati A, Tetzlaff J, Altman DG, The PRISMA Group (2009). Preferred Reporting Items for Systematic Reviews and Meta‐Analyses: The PRISMA Statement. PLoS Med 6(7): e1000097. https://doi.org/10.1371/journal.pmed1000097

After removing duplicates, 3576 potentially relevant citations were found from library databases (131 from other sources, Fig. 1), 30 full texts were reviewed, one study was ongoing 32, 12 studies were included in the review and four were meta‐analysed. Table 1 gives the studies’ characteristics, including the numbers of participants providing longitudinal data and hence which could potentially be aggregated in a meta‐analysis. This was not always the total number of study participants; for example, from RCTs, only women randomized receiving NRT could provide such data. Two study reports contained sufficient data for inclusion in meta‐analysis 29, 30. For another two, authors re‐analysed their data to provide sufficient information 27, 39, 40. Eight studies were reported narratively; for one of these, the authors provided sufficient extra data for a ‘within‐person’ mean difference in urinary cotinine values to be calculated; this could not be combined with values obtained from saliva assays, however 38. For the seven remaining narratively reported studies, mean differences were calculated by subtracting published group mean cotinine or nicotine levels when abstinent and using NRT from those measured when smoking, ignoring between‐participant variability. In two of these seven studies, only some followed‐up women were abstinent and using NRT and these women could not be identified from other study participants 22, 37.

**Table 1:** Characteristics of included studies.

Study	Participants potentially providing longitudinal data (n)	Characteristics of participants		Setting, design & exposures	Body fluid used and assay timings	Assay results
**Cohort studies**						
Gennser, Maršál & Brantmark 1975 33	12	Values are ranges Maternal age 20–31 years^a^ Gestational age 33–39 weeks^a^ Daily cigarette consumption 7–20^a^		Sweden Laboratory‐based longitudinal cohort study; not a smoking cessation intervention study One of each below exposure was used on consecutive days: • 1 ‘standard’ cigarette: talking not permitted and deep inhalation encouraged; average smoking time was 5 minutes • 2 mg gum chewed for 30 minutes • 4 mg gum chewed for 30 minutes	Blood nicotine 5 and 30 minutes after starting to smoke a cigarette 30 and 60 minutes after starting to chew 1 piece of gum	Mean ± SEM (ng/ml) Smoking (*n* = 12) 5 minutes 41.3 ± 3.5 30 minutes 19.6 ± 1.4 2 mg gum (*n* = 6) 30 minutes 10.4 ± 0.6 60 minutes 10.0 ± 0.7 4 mg gum (*n* = 6) 30 minutes 17.5 ± 1.3 60 minutes 14.7 ± 1.3
Oncken *et al*. 1996 34	15	Values are means ± SD Maternal age 28 ± 6^b^ Weight (lb) 169 ± 29^b^ Gestational age 28.1 ± 3.2^b^ Daily cigarette consumption 19 ± 6^b^ Fagerström value 5.7 ± 2^b^		United States Longitudinal cohort study; not a smoking cessation intervention study Women allocated to receive a 5‐day course of 2 mg NRT gum and advised to use ≥ 6 pieces/day but < 30 pieces/day and ≤ 2 pieces/hour Observed chewing to ensure proper technique on first use Reported daily adherence (mean ± SD) Day 1, 6 ± 3 pieces, day 2, 9 ± 5, day 3, 8 ± 3, day 4, 8 ± 3, day 5, 5 ± 2 (last day was only a half‐day)	Blood cotinine 30 minutes after last cigarette After 5 days using nicotine gum while remaining abstinent Also presented trough and peak nicotine levels after smoking one cigarette and chewing 1 piece 2 mg gum	Mean ± SD (ng/ml) Smoking (*n* = 15) 153 ± 18 5 days gum (*n* = 15) 33 ± 8 1 cigarette Trough 6.7 ± 0.8 Peak 19.7 ± 1.5 1 piece 2‐mg gum Trough 3.3 ± 0.5 Peak 5.7 ± 0.7
Oncken *et al*. 1997 35	15	Values are means ± SD Maternal age 28 ± 5.4 Gestational age 28 weeks, 3 days ±20 days Height 161 ± 5.9 cm Weight 63.3 ± 9.2 kg Daily cigarette consumption 20.2 ± 5.2 Values are *n*s Ethnicity data White 10 Black 2 Hispanic 3		United stated Laboratory‐based longitudinal cross‐over study; not a smoking cessation intervention study Two groups of women used Both groups: Smoked *ad libitum* for 8 hours Abstinent for 13 hours before placement of a 21‐mg transdermal nicotine patch for 8 hours They were randomized to either smoking or patch use in session 1 and then a week later crossed over to the other experimental condition	Blood nicotine Hourly assays when smoking Assays at 2 3, 4, 6 & 8 hours after placement of nicotine patch Maximum mean nicotine concentration, time to reach max. and the mean area plasma nicotine concentration‐time curve were reported in the text of this paper	Mean ± SD (ng/ml) Maximal plasma level smoking 19.7 ± 8.09 Patch 16.0 ± 3.5 Time to reach max Smoking 5 hours ± 2.4 Patch 3.2 hours ± 1.7 Area under the plasma Nicotine concentration time curve Smoking 89 ng hour/ml Patch 93 ng hour/ml Mean difference ± SEM 4.8 ± 10.3 ng hour/ml
Wright *et al*. 1997 23	6	Values are means & ranges Maternal age 25.7 (21–31) Weight 82.05 kg (66.1–87.5 kg) Gestational age 34.2 (28.1–37) All white participants Daily cigarette consumption ½–2 packs		United States Laboratory‐based cohort study; not a smoking cessation intervention study Smoking as normal prior to starting study Abstinent for 11 hours prior to patch placement. Transdermal patch 21 mg worn for 8 hours	Saliva nicotine and cotinine Minimum 1 hour after last cigarette 8 hours after of started patch	Mean, range (μg/l)^c^ Cotinine values (*n* = 6) Smoking 100 (40–155) Patch 55 (20–100) Nicotine values (*n* = 6) smoking 19 (7–48) Patch 19 (6–41)
Ogburn *et al*. 1999 36	21	Values are means ± SD Maternal age 26.5 ± 5.7 Gestation at enrolment 27.4 ± 2.7 Daily cigarette consumption 20.5 ± 8.7		United States Laboratory‐based cohort study; not a smoking cessation intervention study Smoking as usual prior to day of admission Abstinence started on admission and continued for in‐patient period Transdermal patch 22 mg/24 hours worn each day of in‐patient stay	Blood nicotine and cotinine At 2 p.m. after a normal morning's smoking and at least 10 minutes after last cigarette On all of days 1–4, 8 hours after patch placement on each day	Mean ± SD (ng/ml) Cotinine values Smoking 116 ± 54 (*n* = 21) Day 1 patch 142 ± 47 (*n* = 21) Day 2 patch 128 ± 38 (*n* = 20) Day 3 patch 123 ± 42 (*n* = 20) Day 4 patch 117 ± 38 (*n* = 20) Nicotine values Smoking 14.4 ± 9.7 (*n* = 20) Day 1 patch 12.7 ± 4.2 (*n* = 21) Day 2 patch 12.8 ± 4.5 (*n* = 20) Day 3 patch 13.7 ± 6.0 (*n* = 20) Day 4 patch11.8 ± 3.9 (*n* = 20)
Hegaard, Kjærgaard, Møller, Wachmann & Ottesen 2004 29	75	Values are means ± SD Gestation at enrolment 21.5 ± 8.4^b^ Daily cigarette consumption 12.5 ± 5.2^b^ Values are ns		Denmark Longitudinal cohort study; smoking cessation intervention study Smoking as normal prior to study Fagerström score used to allocate NRT; higher score, higher dose used and women followed up in 3 parallel groups Women with Fagerström score of 2–4 used up to 12 pieces of 2 mg gum daily For scores of 4–7, 15 mg/16‐hour nicotine patches were used For scores of 7–10, 15 mg/16‐hour nicotine patches plus up to 8 daily pieces of 2 mg gum were used	Saliva cotinine Baseline measurement taken before starting NRT, while still smoking but no time from last cigarette given 1–2 weeks after smoking cessation while using NRT and abstinent; no time from last gum or from patch placement given	Mean ± SD (ng/ml) 2 mg gum (*n* = 6) Smoking 132 ± 95 NRT 35 ± 28 Mean difference, 95% CI = –97 (−6–200) Smoking 173 ± 41 NRT 70 ± 33 Mean difference, 95% CI = –103 (60–146) Patch 15 mg/16 hours, 2 mg gum (*n* = 5) Smoking 246 ± 91 NRT 105 ± 51 Mean difference, 95% CI = –141 (47–236)
		Fagerström value	n			
		2–4	6			
		4–7	7			
		7–10	5			
Oncken, Campbell, Chan, Hatsukami & Kranzler 2009 30	14	Values are means ± SD Maternal age Patch 29.86 ± 6.52 Spray 30.29 ± 5.09 Gestation at enrolment Patch 32.06 ± 2.64 Spray 31.70 ± 3.2 Daily consumption of cigarettes Patch 19.64 ± 3.66 Spray 16.71 ± 5.90 Fagerström value Patch 6.71 ± 1.60 Spray 6.29 ± 1.11 Ethnicity data Caucasian patch 85.7%; spray 71.4%		United States Longitudinal cohort study; smoking cessation intervention study Women smoked 7 cigarettes over 7 hours (1 per hour) in a monitoring session Then they were allocated to one of two types of NRT for 4 days, or the placebo equivalent. They were not monitored during the 4 days and then returned for a 2nd monitoring session NRT used: Transdermal patch 15 mg/16 hours (1 patch per day). During monitoring session patch was placed at 10 a.m. Nasal spray (1 mg/dose) dose = 1spray to each nostril; instructed to use up to 24 daily doses. During monitoring session nasal spray was used twice at the equivalent times to cigarette 1, 4 and 7, and once in place of the 2nd, 3rd, 5th and 6th cigarette	Serum nicotine & cotinine (cotinine used in analysis) 1st laboratory‐based smoking session; after overnight (8 hours) abstinence. Assays taken before and after 1st and 7th cigarette and after 4th cigarette; average of these samples reported 2nd laboratory session: on the 5th treatment day, after overnight NRT abstinence. In both patch and spray groups samples were taken at equivalent times to the smoking session; average of these samples reported	Mean ± SD (ng/ml) Patch (*n* = 7) smoking 138 ± 55 Patch 75 Mean difference ± SD −63 ± 33 Spray (*n* = 7) Smoking 130 ± 57 NRT session spray 39 Mean difference ± SD −91 ± 38
**Randomized controlled trial data**						
Wisborg, Henriksen, Jespersen & Secher 2000 37	124 randomized to nicotine	Values are means ± SD Maternal age 28.2 ± 4.9^b^ Daily cigarette consumption 13.4 ± 4.0^b^		Denmark Randomized controlled trial study; smoking cessation intervention study; recruited from Aarhus Hospital Women randomized to NRT were issued with 8 week's 15 mg/16 hours nicotine patches, followed by a further 3 weeks of 10 mg/16 hours nicotine patches	Saliva cotinine While still smoking usual amount: no time from last cigarette reported At 8 & 11 weeks from starting nicotine patch Further assays at 4 weeks before expected delivery, but unclear whether NRT was still supplied	Mean ± SD (ng/ml) Smoking (baseline) (*n* = 124) 231 ± 125 8 weeks^d^ patch (*n* = 90) 153 11 weeks^d^ patch (*n* = 83) 121 4 weeks^d^ pre‐delivery patch (*n* = 75) 120
Oncken *et al*. 2008 38	100 randomized to nicotine	Values are means ± SD Maternal age 25.5 ± 6.8^b^ Gestation at enrolment 17.1 ± 5.6^b^ Daily consumption of cigarettes before pregnancy 17.5 ± 9.6^b^ last 7 days before enrolment 10.2 ± 6.6 Fagerström value 3.83 ± 1.91^b^ Values are *n*s Ethnicity data^b^		United States Randomized controlled trial; smoking cessation intervention study; recruited from Hartford, New Britain, Springfield medical sites Women randomized to NRT arm were encouraged to stop smoking or to reduce the number of cigarettes smoked If quitting they were instructed to chew one piece of gum for every cigarette they usually smoked per day and to begin on their quit date. If not abstinent they were to chew one piece of gum for each cigarette eliminated; not exceeding 20 pieces/day They were given gum for 6 weeks to use as above and for a further 6 weeks to taper the amount used and stop	Urine cotinine While still smoking usual amount: no time from last cigarette reported 6 weeks after starting gum & 32–34 weeks gestation Further measurements taken at 3–7 days, 2 weeks & 3 weeks after quit date, and 6–12 weeks postpartum but values not reported	Mean ± SD (ng/ml) Smoking (baseline) (*n* = 93) 672 ± 438 6 weeks^d^ of 2 mg gum (*n* = 51) 542 ± 454 32–34 weeks^d^ gestation after NRT treatment finished (*n* = 54) 492.45 ± 443
		Hispanic	53			
		Non‐Hispanic white	38			
		Non‐Hispanic black	8			
		Other	1			
El‐Mohandes *et al*. 2013 22	26 randomized to nicotine	Values are means ± SD Maternal age 27.5 ± 5^b^ Gestation at enrolment 19.6 ± 5.1^b^ Ethnicity data^b^ All self‐report as a minority ethnicity BMI 28.1 ± 10.7^b^ Daily cigarette consumption 7 ± 7.4^b^ Values are *n*s Amount of cigarette		United States Randomized clinical trial; smoking cessation intervention study; recruited from prenatal care sites in the DC metropolitan area Women randomized to NRT used transdermal 14‐mg patch for approx. 2–4 weeks before first sampling, followed by transdermal 7 mg patch for approx. 2 weeks before the second sampling Some women received 21 mg patch for first 2 weeks of NRT due to higher baseline cotinine levels but would have been on 14 mg patch at time of first sampling. Samples were also taken later when it is unclear if women were still given NRT	Saliva cotinine While still smoking usual amount: no time from last cigarette reported After using a 14 mg patch for approx. 2‐4 weeks (and having been using NRT for average 3.7 weeks) After using a 7 mg patch for approx. 2 weeks (and having been using NRT for average 7.8 weeks)	Mean ± SD (ng/ml) Smoking (baseline) (*n* = 26) 171 ± 143 14 mg patch^d^ (*n* = 26) 142^c^ 7 mg patch^d^ (*n* = 26) 129^c^
		Smoke all of a cig.	7			
		Smoke most of a cig.	3			
		Smoke half a cig.	15			
		Inhalation Inhale deeply	8			
		Inhale moderately	10			
		Inhale slightly	8			
Berlin, Grangé, Jacob & Tanguy 2014 39	203 randomized to nicotine	Values are median & interquartile range Maternal age 29.1 (25–34)^b^ Gestation at randomization 17 (15–20)^b^ BMI pre‐pregnancy 23 (20–27)^b^ Daily cigarette consumption 11 (8–15)^b^ Fagerström value 5 (3–6)^b^ Values are *n*s Ethnicity data^b^ European 194 African 6 Asian 1 Other 2 Time to first cigarette^b^ > 60 minutes 31 31–60 minutes 36 6–30 minutes 74 ≤ 5 minutes 62		France Randomized controlled trial; smoking cessation intervention study; recruited from maternity wards Nicotine patch between 10–30 mg/day adjusted to participant cotinine level (mean ± SD = 18 ± 6.8 mg/day) used from quit date until delivery with brief smoking lapses permitted	Saliva cotinine While still smoking usual amount: no time from last cigarette reported 2 weeks after quit date while using nicotine patch 8 weeks after quit date while using nicotine patch	Mean ± SE (μg/l) Smoking (baseline) 119 ± 1.09 2 weeks patch^d^ 108 ± 1.1 8 weeks patch^d^ 80 ± 1.1 Unclear how many women sampled at each visit
Bowker, Lewis, Coleman, Vaz & Cooper 2014 40	33	Values are median & interquartile range Maternal age 26.12 (22.29–32.35) Gestation at baseline 14.4 (13.3–17.8) Ethnicity data All white except one Asian/other BMI 25.6 (22.7–29.3) Women with partners who smoke 23 (70%) Heaviness of smoking index 3 (2–3) Values are *n*s Number of cigarettes smoked 5–10 cigarettes (22) 11–15 (8) >20 (3)		United Kingdom Secondary analysis of data from intervention arm of randomized controlled trial of NRT; smoking cessation intervention study; recruited from antenatal clinics Transdermal patch 15 mg/16 hour used for up to 2 months after quit date while remaining abstinent; instructed to stop NRT if smoking re‐starts	Saliva cotinine While still smoking usual amount: no time from last cigarette reported 4 weeks from quit date while using 15 mg/16‐hour nicotine patch and abstinent Reported difference in cotinine from smoking to NRT use when starting Cotinine level > 150 ng/ml and when <150 ng/ml	Median & interquartile range (ng/ml) Smoking (*n* = 33) 98.5 (71.3–177.8) NRT (abstinent) (*n* = 33) 62.8 (33.3–82.7) Median cotinine difference with baseline cotinine: >150 ng/ml −134.8 < 150 ng/ml–27.9

a Values only reported for all participants in the study, not solely women in the longitudinal analysis.

b Values reported for all women enrolled, not only women analysed.

c Data valued obtained using WebPlotDigitizer and SD not available 24.

d Samples taken on all randomized to nicotine in RCT irrespective of smoking status; women could be smoking or abstinent. SEM = standard error of the mean; SD = standard deviation; CI = confidence interval; NRT = nicotine replacement therapy; BMI = body mass index.

### Characteristics of included studies

Seven studies were set in the United States 22, 23, 30, 34, 35, 36, 38, two recruited from Denmark 29, 37 and one each from France 39, United Kingdom 40 and Sweden 33. Seven were longitudinal cohorts designed to investigate the impacts of smoking and then NRT use in the same women 23, 29, 30, 33, 34, 35, 36, and five were either RCTs 22, 37, 38, 39, 40 or presented secondary analyses of RCT data 40. Studies tested 2 mg nicotine gum 29, 33, 38; 4 mg gum 33; 7 mg/24‐hour1Not all papers explicitly reported patch duration; where necessary this has been derived from knowledge of available nicotine patches. nicotine patch 22; 14 mg/24‐hour nicotine patch 22; 15 mg/16‐hour nicotine patch 29, 30, 37, 40; 15 mg/24‐hour nicotine patch plus 2 mg gum (often called ‘dual NRT’) 29; 21 mg/24‐hour nicotine patch 23, 35; 22 mg/24‐hour patch 36; nicotine nasal spray 30; and also individualized nicotine dosing based on saliva cotinine levels 39. Three papers reported studies recruiting relatively early in pregnancy; two RCTs reported women's mean gestational age at enrolment as 17 weeks 38, 39 and another as 14 weeks 40. All except one of the remaining studies (22 weeks) 29 reported mean gestations at recruitment of 27 weeks or later. Timings for body fluids sampling while using NRT varied greatly; in laboratory (or in‐patient) studies sampling occurred within 1 hour 33; at 30 minutes and 5 days 34; at up to 8 hours 23, 35; during a 4‐day period 36; and at both 8 hours and 4 days 30. In non‐laboratory studies (mainly RCTs), sampling occurred at 1–2 weeks 29, 8–11 weeks and also 4 weeks before delivery 37; 6 weeks 38; 2–4 weeks 22, 4 weeks 40; and at both 2 and 8 weeks after starting NRT 39.

### Quality assessment

Quality assessments are reported in Table 2. The seven longitudinal cohort studies were of variable quality; six were awarded three or more stars out of seven. Studies used appropriate biochemical validation methods and generally scored well on follow‐up completeness, but they scored less strongly with regard to the timing of samples when smoking or using NRT or in how abstinence was confirmed before or while using NRT, usually due to lack of detail in study descriptions.

**Table 2:** Quality assessment of cohort studies.

Study	Representativeness	Ascertainment of exposures	Method for confirming abstinence	Appropriateness sample timing	Completeness of follow‐up	Stars (out of 7)
Gennser 1975 33	–	*	*	*	**	5*
Oncken 1996 34	**	*	*	–	*	5*
Oncken 1997 35	**	–	*	–	**	5*
Wright 1997 23	–	–	*	–	**	3*
Ogburn 1999 36	*	–	*	–	*	3*
Hegaard 2004 29	*	–	*	–	–	2*
Oncken 2009 30	**	*	*	*	**	7*

### Studies’ findings

#### Meta‐analysis

**Figure 2:**
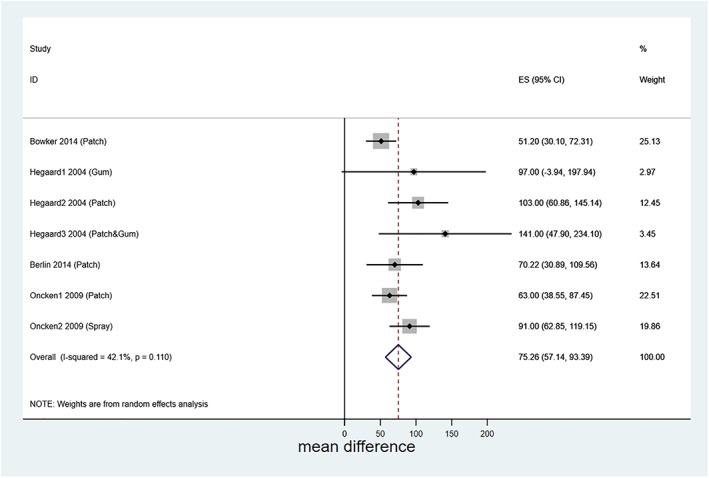
Forest plot showing meta‐analysis of mean difference in saliva cotinine levels when smoking and when abstinent but using nicotine replacement therapy (NRT). Hegaard1, Hegaard2 and Heggard3 2004 represent cohorts of women given different forms of NRT and reported in Hegaard 2004 29; ditto for Oncken1 and Oncken2 2009 and Oncken 2009 30. [Colour figure can be viewed at wileyonlinelibrary.com]

Data obtained from 83 participants in four saliva cotinine‐measuring studies were included in a meta‐analysis (Fig. 2; Table 3, rows 1–4) 29, 30, 39, 40. The pooled estimate for the mean difference (95% CIs) between saliva cotinine levels when smoking and when using NRT and abstinent from smoking was 75.3 (57.1–93.4) ng/ml (*I* ^2^ = 42.1%, *P* = 0.1). Within these studies, percentage nicotine substitution varied between 26.5% (2 mg gum) 29 and 60.0% (15 mg/16‐hour patch).

**Table 3:** Source and derivation of mean differences used in review.

Studies in meta‐analysis								
Row	Study	Participants abstinent on NRT, providing longitudinal data and loss to follow up (n)	Body fluid & assay times (i.e. after starting NRT)	Type and dose of NRT		Outcomes^b^		Derivation of outcome data
						Mean difference (SEMD)^d^ (ng/ml)	Percentage substitution (%)	
1	Hegaard 2004 29	18 analysed	Saliva cotinine, 2 weeks	2 mg gum (*n* = 6)		97 (51.5)	26.5	Mean differences taken directly from paper SEMs calculated from CI presented in paper
		Of 75 enrolled 40 were excluded:		15 mg/16‐hour patch (*n* = 7)		103 (21.5)	40.5	
		16 dropped out						
		13 stopped NRT		15 mg/16‐hour patch and 2 mg gum (*n* = 5)		141 (47.5)	42.7	
		11 smoked the day before second sample						
		17 were not analysed:						
		15 samples not collected or not treated properly or went missing						
		2 used a 10‐mg patch						
2	Oncken 2009 30	14	Serum cotinine, 4 days	15 mg/16‐hour patch (*n* = 7)		63 (12.47)	54.3	Mean differences taken directly from paper
				Spray 24 dose/day = 24 mg/24 hours (*n* = 7)		91 (14.36)	30.0	SEM calculated from SD of difference
3	Bowker 2014 40	33	Saliva cotinine, 4 weeks	15 mg/16‐hour patch		51.2 (10.77)	60.0	Mean difference and SEM calculated using original study data
4	Berlin 2014 39	18	Saliva cotinine, 2 weeks^c^	Patch, variable strength		70.22 (20.07)	49.3	Mean difference and SEM of abstinent women only calculated by study author using original study data

Narratively reported studies								
Studies with additional data from authors								
Row	Study	Participants abstinent on NRT, providing longitudinal data and loss to follow‐up (n)	Body fluid & assay times (i.e. after starting NRT)	Type and dose of NRT	Outcomes^b^		Derivation of outcome data/reason for non‐inclusion in meta‐analysis	
					Mean difference (SEMD)^d^ (ng/ml)	Percentage substitution (%)		
5	Oncken 2008 38	4	Urine cotinine, 6 weeks	2 mg gum (*n* = 4)	130.00 (245.738)	65.2	Mean difference and SD of abstinent women using gum at 6 weeks calculated by author from original data	
							Not included in meta‐analysis as urine rather than saliva cotinine value	

Studies where only published data used								
Row	Study	Participants abstinent on NRT, providing longitudinal data and loss to follow‐up (n)	Type & dose of NRT body fluid & assay times (i.e. after starting NRT)	Outcomes^b^ (ng/ml unless stated otherwise)			Reasons for non‐inclusion in meta‐analysis	
6	Gennser 1975 33	Unclear: 12 participants smoked at baseline, 6 were later using NRT but it is not clear which of the original 12 these were	Gum, 2 & 4 mg Blood nicotine, 30 minutes	Mean ± SEM			Only group means and their standard errors presented in paper; standard error of mean difference could not be calculated	
				Cig. 19.6 ± 1.4 (n = 12)				
				2 mg gum 10.0 ± 0.7 (*n* = 6)				
							Difference in means calculated by review team as (group mean value for smoking) – (group mean value for gum use)	
				Difference in means = 9.6				
				Cig. 19.6 ± 1.4 (*n* = 12)				
				4 mg gum14.7 ± 1.3 (*n* = 6)				
				Difference in means = 4.9				
7	Oncken 1996 34	15	Gum, 2 mg blood cotinine, 5 days	Mean ± SD			Only group means and their standard errors presented in paper; standard error of mean difference could not be calculated	
		Of 19 enrolled 4 dropped out: 2 due to non‐abstinence 2 suffered severe nausea						
				Smoking 153 ± 18				
				5 days gum 33 ± 8				
				Difference in means = 120			Difference in means calculated by review team as (group mean value for smoking) – (group mean value for gum use)	
8	Oncken 1997 35	15	Patch, 21 mg blood nicotine/hour	Mean ± SD (overlap: smoking group lower cotinine than patch group)			Only group means and their standard errors presented in paper; standard error of mean difference could not be calculated	
		Of 17 enrolled 2 were excluded: 1 single uterine artery 1 quit smoking after session 1						
							Difference in maximal plasma level calculated as (smoking maximal level) – (patch maximal level)	
				Mean maximal plasma level				
							Mean difference of areas under the plasma nicotine concentration time curve taken directly from paper	
				Smoking 19.7 ± 8.09				
				Patch 16.0 ± 3.5				
				Difference in mean maximal plasma level 3.7				
				Time to reach max.				
				Smoking 5 hours ± 2.4				
				Patch 3.2 hours ± 1.7				
				Area under the plasma nicotine concentration time curve				
				Smoking 89 ng‐hour/ml				
				Patch 93 ng ‐hour/ml				
				Mean difference ± SEM of area under the plasma nicotine concentration time curve − 4.8 ± 10.3 ng‐hour/ml				
9	Wright 1997 23	6	Patch, 21 mg saliva cotinine, 8 hours	aMean & range (μg/l)			Only group means and their standard errors presented in paper; standard error of mean difference could not be calculated	
				Smoking 100 (40–155)				
				Patch 55 (20–100)				
				Difference in means = 45				
							Difference in means calculated by review team as (group mean value for smoking) – (group mean value for gum use)	
10	Ogburn 1999 36	21 no reason given for missing data	Patch, 21 mg blood cotinine, day 1–4	Mean ± SD			Only group means and their standard errors presented in paper; standard error of mean difference could not be calculated	
				Cotinine values				
				Smoking 116 ± 54 (*n* = 21)				
				Day 1 patch 142 ± 47 (*n* = 21)				
				Day 2 patch 128 ± 38 (*n* = 20)				
				Day 3 patch 123 ± 42 (*n* = 20)				
				Day 4 patch 117 ± 38 (*n* = 20)			Difference in means calculated by review team as, (group mean value for smoking) – (group mean value for gum use); missing data ignored	
				Difference in means				
				Days 1–26				
				Days 2–12				
				Days 3–7				
				Days 4–1				
11	Wisborg 2000 37	Unclear, as not all participants were abstinent when assays taken	Patch 15 mg, 10 mg Saliva cotinine, 8 & 11 weeks	Mean ± SD			Not all participants abstinent when assays occurred; only cross‐sectional data available in published report	
				Smoking (*n* = 124) 231 ± 125 8 weeks (15 mg) patch (*n* = 90)				
				153 Difference in means at 8 weeks, 78				
				11 weeks (10 mg) patch (*n* = 83) 121. Difference in means at 11 weeks, 110				
12	El‐Mohandes 2013 22	Unclear, as not all participants were abstinent when assays taken	Patch, 14 mg, 7 mg Saliva cotinine, approx.2–4 & approx. 4–6 weeks	Mean ± SD (ng/ml) (*n* = 26)			Not all participants abstinent when assays occurred; only cross‐sectional data available in published report	
				Baseline 171 ± 143 14‐mg patch^a^ 142. Difference in means 39				
							Difference in means calculated by review team as, (mean value for patch use) – (mean value for smoking)	
				Baseline 171 ± 143 7‐mg patch^a^ 129 Difference in means 42				

a Data valued obtained using WebPlotDigitizer 24.

b A negative mean difference/difference between means indicates higher cotinine/nicotine levels when smoking.

c Data from one of two follow‐up times selected to avoid inclusion of non‐independent observations in meta‐analysis.

d Standard error of the mean (SEM) difference. SD = standard deviation; CI = confidence interval; NRT = nicotine replacement therapy; BMI = body mass index.

#### Narratively reported studies

In six of the eight narratively reported studies, irrespective of body fluid (or substance assayed), exposure levels were higher when smoking than when abstinent and using NRT. In the remaining two studies, findings were mixed and details follow; Table 3 shows mean differences and explains which data were used to derive these and reasons for exclusion from meta‐analysis. Also, numbers of participants for whom longitudinal data were available are given and, as relevant, how these related to total study samples.

The study summarized in row 5 reported higher urinary cotinine levels when smoking 35. Although longitudinal data were available, findings from this study could not be used for the meta‐analysis as other studies in this analysis reported saliva cotinine.

In rows 6–9, four longitudinal cohort studies are described 23, 33, 34, 35; in three, exposure measured as nicotine or cotinine was higher when smoking 23, 33, 34. The fourth 35 had inconsistent findings; peak exposure (mean maximal plasma nicotine) was higher but total exposure (area under a nicotine concentration versus time graph) was lower after smoking.

Row 10 describes a longitudinal cohort study in which women were followed‐up daily for 4 days when abstinent after starting NRT 36; cotinine levels (Table 3) were higher and nicotine levels (not shown) were lower at all follow‐up points, with the day 1 cotinine difference reaching statistical significance. For three follow‐up comparisons, a participant (from 21) was lost to follow‐up (Table 1).

Rows 11 and 12 describe women in NRT arms of RCTs 22, 37; in both studies, exposures (group mean cotinine levels) were higher in smokers at baseline, but it was not possible to identify separately those using NRT and abstinent.

## Discussion

A meta‐analysis comparing cotinine exposures when pregnant women smoke with those when they use NRT found that levels were, on average, 75.3 ng/ml lower when abstinent and using NRT than when the same women smoked. Similarly, lower exposures after NRT occurred in six of the remaining eight studies.

Only 12 empirical studies were included; five had not been designed as longitudinal cohorts and most did not publish sufficient details to be included in a meta‐analysis. Nevertheless, longitudinal, within‐participant data were available from 10 studies and so only two were of limited use for answering review questions 22, 37. Participants were recruited to either hospital in‐patient/laboratory studies with intensive protocols or into clinical trials, but the consistency of outcomes from studies in very different settings suggests the principal finding that using NRT exposes pregnant women who are fully abstinent from smoking to less nicotine than smoking is valid. Although the amount of useable data from studies was small, by focusing on ‘within‐individual’ differences in cotinine levels, study women effectively acted as their own controls and external impacts on cotinine levels, apart from of NRT doses used, were eliminated. Only factors which changed within individual women between baseline and follow‐up could be expected to affect the pooled estimate for mean difference in cotinine levels. One such factor is the rate of nicotine metabolism, which is significantly accelerated by the second trimester 15. Adjusting findings for increasing rates of nicotine metabolism as pregnancy progressed could have helped us to understand how much lower cotinine levels on NRT might be attributable to faster metabolism; however, this was beyond the scope of the review. Nevertheless, there are two reasons to suspect that increased nicotine metabolism had little overall impact on findings. First, the mean differences from studies which measured these only hours after stopping smoking 23, 34, 35 were comparable to those in whom cotinine (nicotine) levels on NRT were measured weeks afterwards or even later in pregnancy 22, 37, 38, 39, 40. Secondly, findings from those studies which recruited more women who were under 18 weeks gestation 38, 39, 40 appeared similar to remaining studies which recruited later in pregnancy.

We believe this study is original, and the systematic approach used combined with the rigorous contact made with authors should have sourced all available data within identified studies. Despite substantial variation in the types of NRT issued and in how participants were instructed to use this, and also in the timings of sample measurement across studies, the low level of heterogeneity in the pooled mean difference estimate indicates that the data synthesis undertaken was valid and the estimate is robust.

It was not possible to combine studies’ findings to investigate the impacts of different NRT doses or regimens on cotinine levels. However, consideration of individual studies’ findings does not suggest that different NRT doses or giving different instructions about using NRT has substantial impact. For example, the mean differences in cotinine levels obtained when smoking and later from women who were abstinent and used NRT, and so were adherent, were similar in two major RCTs investigating NRT in which participants were told to use this treatment in different ways 39, 41. In one trial 41, a single nicotine patch dose was provided for only an 8‐week treatment course and participants were instructed to remove patches during smoking lapses. In the other trial, however, nicotine patch doses were personalized, and there was potential for higher doses to be delivered to women who were told that they could continue using NRT during brief smoking lapses and even for the whole of pregnancy, if desired 39. The meta‐analysis showed that cotinine levels when abstinent and on NRT were reduced, on average, by 70.3 ng/ml compared to smoking, and throughout the four meta‐analysis studies cotinine levels when smoking varied between 99 and 246 ng/ml, suggesting that reductions in nicotine exposure while using NRT are clinically meaningful. Review studies, SNIPP excepted 39, used standard rather than higher doses of nicotine patches and these delivered no more than 15 mg cotinine in 16 hours or the 24‐hour equivalent. An important, unequivocal message is, therefore, that when pregnant smokers become abstinent and adhere with to ‘standard’ doses of NRT they are, on average, exposed to less nicotine than from smoking. One arm of one study delivered both 15 mg/16‐hour nicotine patches and 2 mg gum to five women 29 who had high baseline cotinine levels when smoking [mean (SD)] 246 (91) ng/ml, and the mean difference (95% CI) between this and cotinine levels on NRT was large [mean difference (95% CI)] –141 (47–236). This estimate lacks precision, however, and provides no evidence that higher‐dose NRT might expose women to more nicotine; nevertheless, more studies are needed.

A key reason for this study was to determine whether pregnant smokers who have concerns about the safety of nicotine in pregnancy and which might deter them from using NRT regularly enough and in sufficiently high doses to help them stop smoking could be reassured about its use 18, 40. The review demonstrates clearly that NRT exposes pregnant women to much smaller nicotine doses than smoking and, clearly, pregnant women considering NRT use in pregnancy can be strongly reassured on this point. It was not an aim of this paper to determine whether or not nicotine is harmful to the developing baby; however, the accruing literature suggests that this is not the case. Although rodent studies have suggested that fetal nicotine exposure may cause infant behavioural problems 42, the only RCT of NRT for smoking cessation in pregnancy found that NRT group infants had better developmental outcomes 43. Additionally, large studies of NRT used in routine health care have found no consistent relationship between NRT use in pregnancy and stillbirth 44, 45, congenital abnormalities 46, 47, preterm birth 48, low birth weight 49 or strabismus 50. It seems most probable that most, if not all, the fetal harms caused by smoking in pregnancy are due to other tobacco smoke toxins. Pregnant women should avoid unnecessary toxin exposure and, compared to smoking, NRT both eliminates exposure to numerous tobacco smoke toxins and reduces nicotine exposure. However, NRT also has great potential for improving fetal health and averting adverse pregnancy outcomes by helping some pregnant women to stop smoking. Review findings could, therefore, help to reassure pregnant women about the probable safety of using NRT to maintain smoking abstinence and also about the use of higher‐dose NRT. Although using ‘dual NRT’, an NRT patch and a short‐acting NRT together would generate higher nicotine exposures, ‘standard NRT dose’‐generated nicotine exposure in abstinent pregnant women is so much lower than that from smoking that dual NRT could well also deliver lower nicotine doses than cigarettes. However, ‘dual NRT’ would be more likely to alleviate withdrawal symptoms and so women would probably use this for longer; this may explain why an observational analysis of UK Stop Smoking Services’ routine data found dual NRT but not standard‐dose NRT associated with smoking cessation in pregnancy 51.

## Conclusions

Among pregnant women who quit smoking, standard‐dose NRT generates lower nicotine exposure than smoking. This lower exposure, combined with the very strong likelihood that nicotine is not responsible for the majority of fetal harms caused by tobacco smoke, makes it very likely that relative to smoking, NRT is safer for the fetus than smoking. Additionally, when NRT promotes maternal smoking cessation this is very likely to improve fetal health by reducing adverse pregnancy outcomes.

## Prospero protocol

Prospero protocol registration number: CRD42017081914

## Declaration of interests

I.B. has received honoraria from Pfizer Ltd for talks and participation in advisory board. C.O. has received study medication (nicotine inhaler and placebo) from Pfizer Ltd for an NIH‐funded study of a nicotine inhaler for smoking cessation during pregnancy. C.H., S.L., K.C., R.C., S.C., T.C/‐H. and T.C.; none to declare.

## Supporting information


**Appendix S1** Example search run in MEDLINE.
**Appendix S2** Amended Newcastle‐Ottawa Scale for Cohort Studies: item definitions.
