# Supplementary material for: Comparison of nicotine exposure during pregnancy when smoking and abstinent with nicotine replacement therapy: systematic review and meta‐analysis
Source: Addiction. 2018 Dec 11;114(3):406–24. doi: 10.1111/add.14473 (PMC6590470; doi:10.1111/add.14473)
Supplement: Supplementary file 1 — Appendix S1 Example search run in MEDLINE. Appendix S2 Amended Newcastle‐Ottawa Scale for Cohort Studies: item definitions. [file ADD-114-406-s001.doc]

**Appendix 1 Example search run in MEDLINE**

1. exp pregnancy/
2. pregnan*.ti,ab.
3. matern*.ti,ab.
4. antenatal.ti,ab.
5. prenatal.ti,ab.
6. 1 or 2 or 3 or 4 or 5
7. smoking.ti,ab.
8. smoking cessation.ti,ab.
9. (NRT or nicotine replacement).ti,ab.
10. ((Quit or quitting or stop or stopping or stopped or abstain or abstinen*) and smoking).ti,ab.
11. exp smoking cessation/
12. exp nicotine replacement/
13. exp NRT/
14. 7 or 8 or 9 or 10 or 11 or 12 or 13
15. nicotine.mp. [mp=ti, ab, hw, tn, ot, dm, mf, dv, kw, fx, nm, kf, px, rx, an, ui, sy, tc, id, tm]
16. cotinine. mp. [mp=ti, ab, hw, tn, ot, dm, mf, dv, kw, fx, nm, kf, px, rx, an, ui, sy, tc, id, tm]
17. 15 or 16
18. 6 and 14 and 17
19. ANIMALS/ not (HUMANS/ and ANIMALS/)
20. 18 not 19
21. Limit 20 to yr=“1980 - Current”

**Appendix 2 Amended Newcastle-Ottawa Scale for Cohort Studies: item definitions**

**1 Representativeness of cohort**

Three criteria were assessed: i) enrolling participants from >1 maternity care site / research centre, ii) enrolling women both before and after 18 weeks gestation, iii) not excluding participants on the basis of ethnicity and iv) not excluding any particular age groups (amongst those of child bearing age). Two stars were awarded if 3 or more criteria met, one star for 2 or 1 and no stars were awarded if no criteria were met.

It was thought important to include women with a range of gestations around 18 weeks because nicotine metabolism becomes significantly accelerated after this time and such acceleration would likely affect cotinine levels.(15)

**2 Ascertainment of relevant exposures (i.e. smoking or using NRT)**

It was important for studies to have robust methods for confirming that participants were smoking and also using NRT when abstinent. Three criteria were used, these were that authors reported methods for confirming that participants i) smoked at baseline (e.g. direct observation or self-report), ii) used NRT (e.g. direct observation) and iii) were abstinent when using NRT (e.g. self-report or exhaled CO readings).

One star was awarded if all 3 criteria were met.

**3 Biochemical or observational confirmation of abst**inence

Robust methods for confirming abstinence when using NRT were thought particularly important. Two criteria were judged particularly robust for confirming abstinence: i) direct observation or monitoring of participants using NRT (e.g. as inpatients) and ii) biochemical validation of abstinence.

If either were used, one star was awarded.

**4 Appropriate timing of samples**

It was considered important that body fluid samples should be obtained at a time after smoking or starting NRT such that trough nicotine (cotinine) levels were avoided; we anticipated that non-trough levels would more closely reflect average nicotine (cotinine) levels generated by regular daytime smoking or NRT use.

For ascertainment of exposure to ***cigarette smoking***, a star was awarded if samples were taken within 20 minutes of finishing a cigarette. After stopping smoking the blood nicotine level immediately starts to drop reaching a trough at around 50 minutes (52), but when people smoke more than one daily cigarette they would likely start their next cigarette soon after starting to experience withdrawal and, therefore, would have higher average body nicotine levels than trough levels.

For ascertainment of exposure to nicotine gum, a star was awarded if samples were taken within 30 minutes of finishing chewing as nicotine levels remain elevated for 30-45 of stopping chewing with only a marginal decrease before 30 minutes. (52) For nicotine patches a star was awarded if samples were taken at least four hours after putting on a patch. Nicotine patches generally takes around one hour to reach the blood stream (53) so allowing 4 hours before sampling permits development of a steady state to have developed. For nicotine nasal spray a star was awarded if samples were taken around 30 minutes after administration. Nasal spray nicotine is absorbed faster than other forms of NRT reaching peak venous blood nicotine level 10 minutes after administration (53); however, venous blood concentration falls slowly before troughing.(54)

**5 Adequacy of cohort follow-up**

Stars were awarded as follows. Complete follow up with all subjects accounted for was awarded 2 stars; adequate follow up, <10 participants lost to follow-up or > 10% but a description given of those lost (1 star); inadequate follow up, >10% participants lost to follow-up with no description given of those lost (0 stars) and no information given about follow up (0 stars)
